# Supplementary material for: Bacterial vitamin sharing emerges from a balance between release and uptake
Source: bioRxiv. 2026 Oct 13:2026.09.01.748612. Preprint. [Version 2] doi: 10.64898/2026.09.01.748612 (PMC13684563; doi:10.64898/2026.09.01.748612)
Supplement: Supplementary file 1 [file NIHPP2026.09.01.748612V2-supplement-1.pdf]

5 Supplementary figures

bioRxiv preprint doi: https://doi.org/10.64898/2026.09.01.748612; this version posted October 14, 2026. The copyright holder for this preprint (which was not certified by peer review) is the author/funder, who has granted bioRxiv a license to display the preprint in perpetuity. It is made available under a CC-BY 4.0 International license.

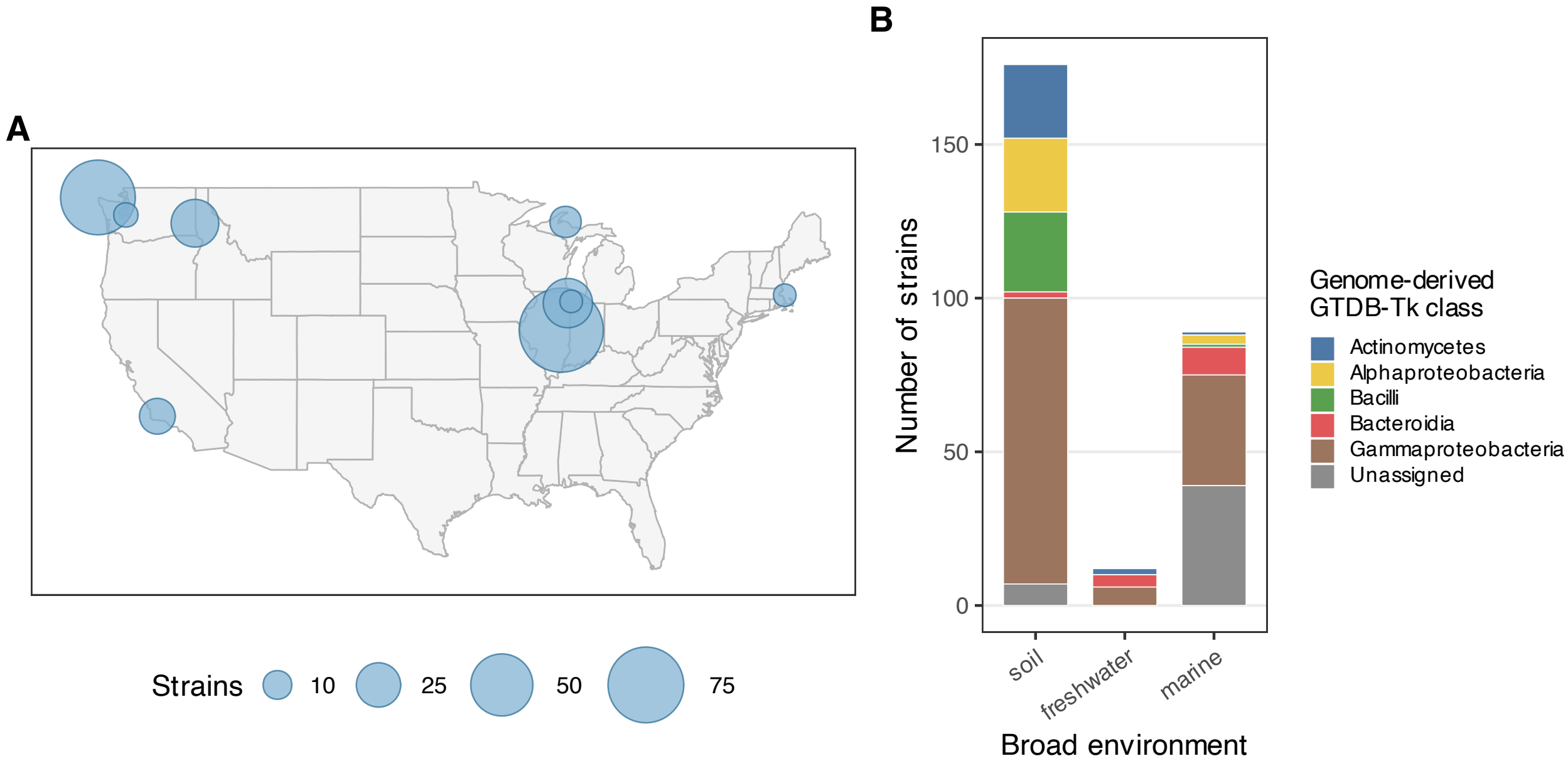

Fig. S1 Geographic, environmental, and taxonomic distribution of the bacterial strain collection. (A) Sampling-location cluster centroids across the continental United States. Sampling coordinates were grouped into ten reproducible clusters; bubble area is proportional to the number of strains assigned to each cluster. (B) Numbers of strains isolated from soil, freshwater and marine environments, stacked by genome-derived GTDB-Tk class. Grey segments denote strains without an assigned GTDB-Tk class.

bioRxiv preprint doi: https://doi.org/10.64898/2026.09.01.748612; this version posted October 14, 2026. The copyright holder for this preprint (which was not certified by peer review) is the author/funder, who has granted bioRxiv a license to display the preprint in perpetuity. It is made available under a CC-BY 4.0 International license.

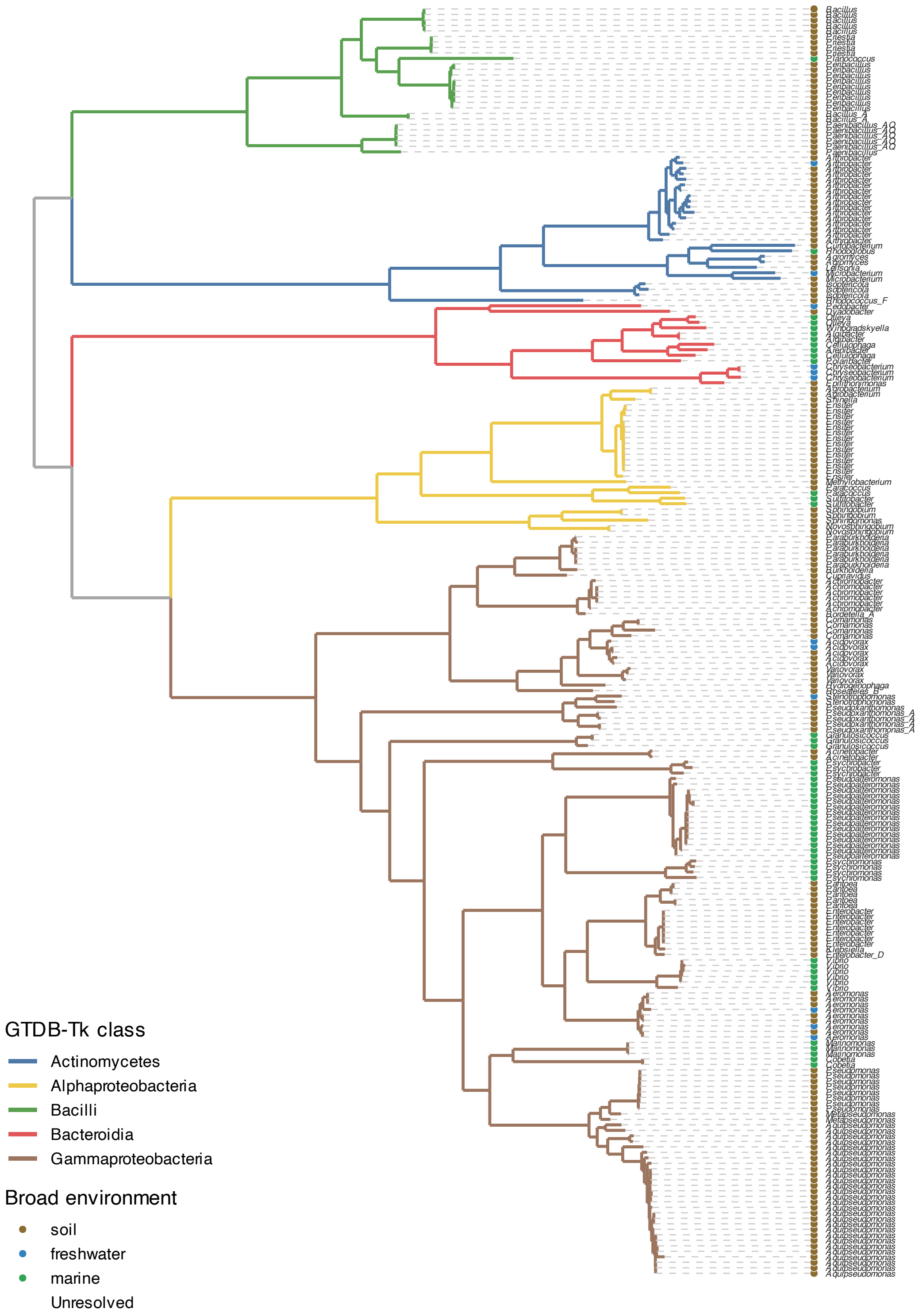

Fig. S2 Phylogenetic relationships among the 231 bacterial genomes retained for phylogenomic analysis. The tree was inferred from aligned bac120 marker genes using FastTree within the GTDB-Tk `de novo_wf` workflow and rooted at the best-supported split between Bacillati and Pseudomonadati. Tip labels indicate GTDB-Tk genus assignments, tip-point colours indicate the broad environments from which the isolates were collected, and branch colours indicate GTDB-Tk class.

bioRxiv preprint doi: https://doi.org/10.64898/2026.09.01.748612; this version posted October 14, 2026. The copyright holder for this preprint (which was not certified by peer review) is the author/funder, who has granted bioRxiv a license to display the preprint in perpetuity. It is made available under a CC-BY 4.0 International license.

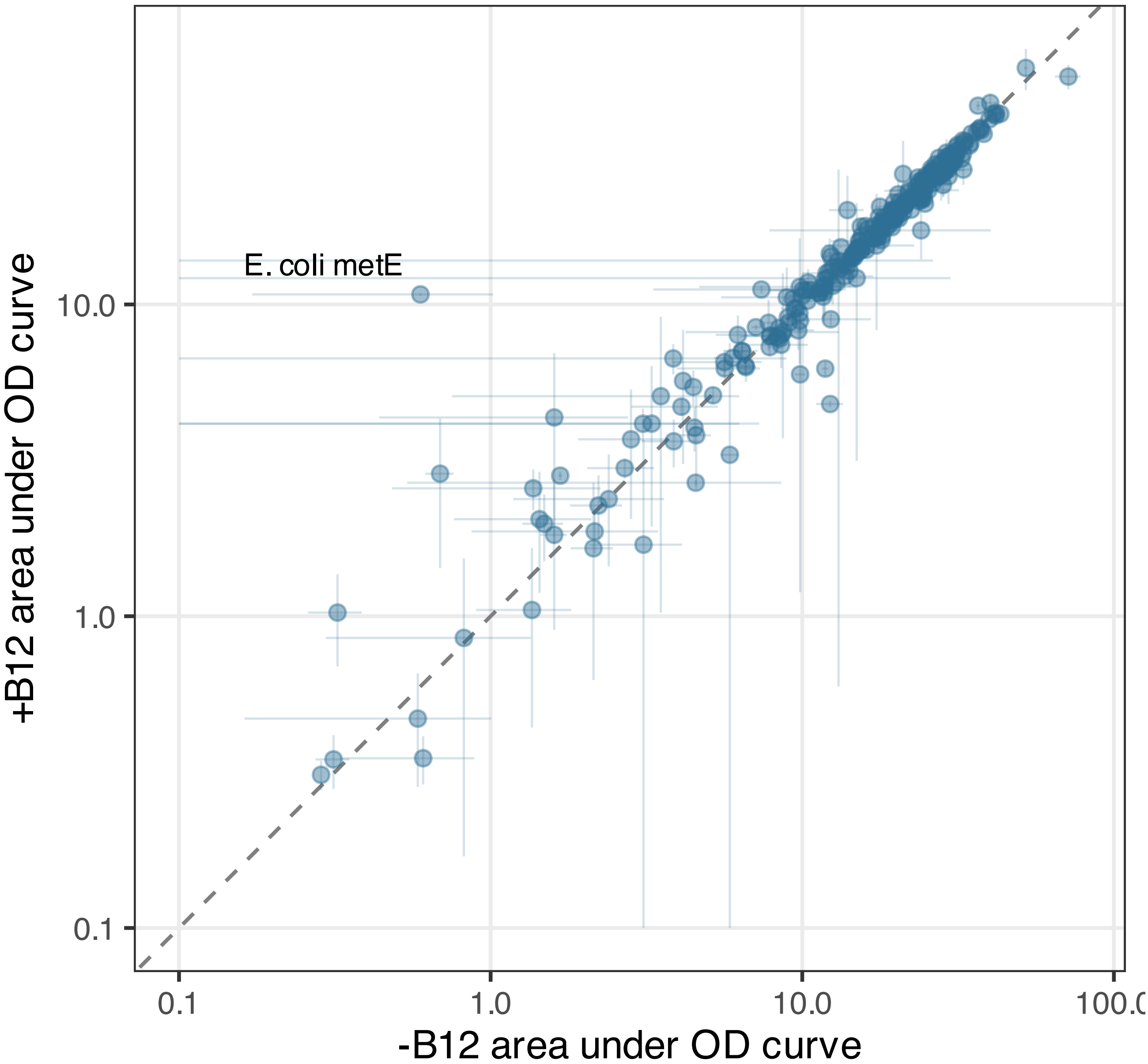

Fig. S3 Growth of 274 bacterial collection isolates and controls in Tm-mod medium with (y-axis) or without (x-axis) 10nM added B_{12}. Growth was assessed as the area under the optical density at 600nm curve over 57 hours with measurements every 93 minutes. The B_{12}- or methionine-dependent *E. coli metE* reporter strain was the only strain that showed substantial deviation from equal growth with and without B_{12} (1:1 diagonal dashed line).

bioRxiv preprint doi: https://doi.org/10.64898/2026.09.01.748612; this version posted October 14, 2026. The copyright holder for this preprint (which was not certified by peer review) is the author/funder, who has granted bioRxiv a license to display the preprint in perpetuity. It is made available under a CC-BY 4.0 International license.

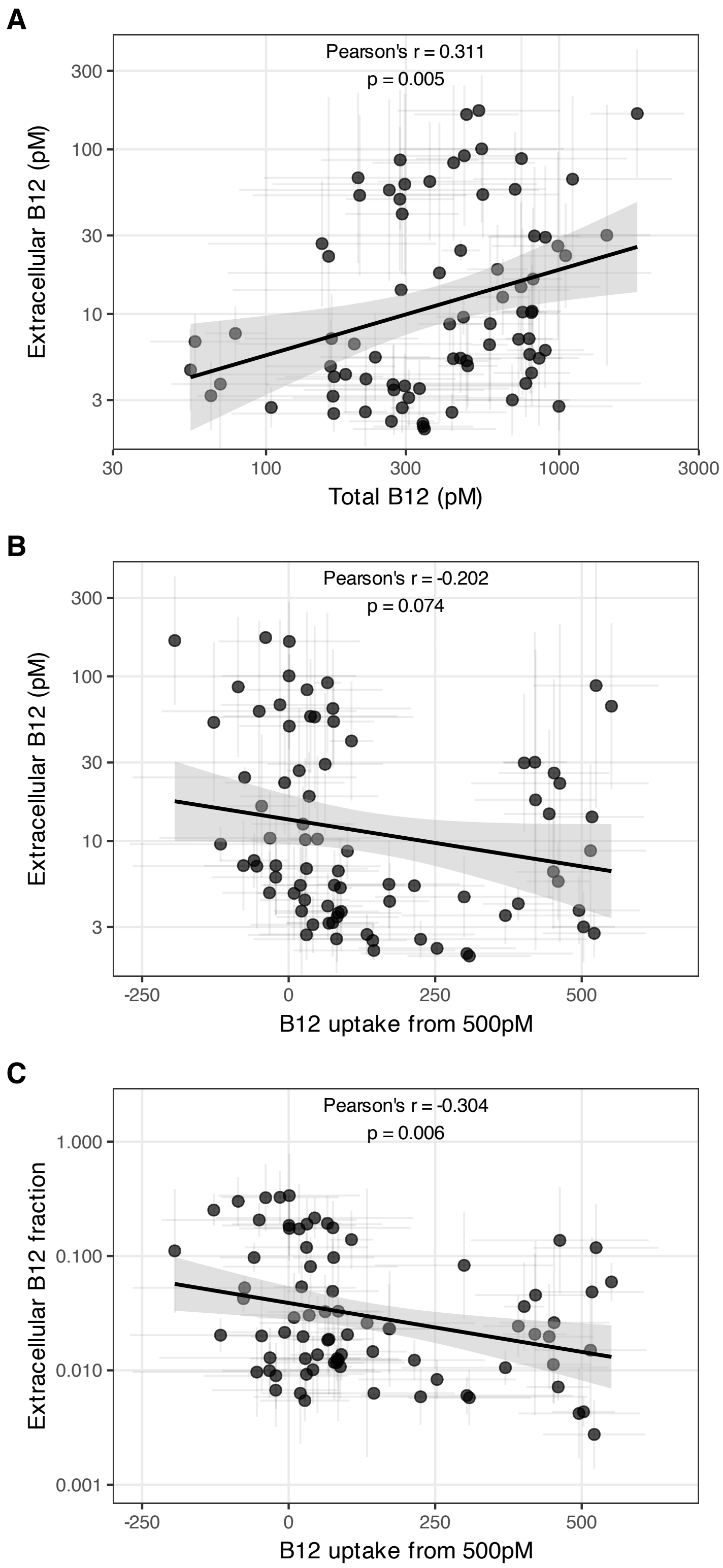

Fig. S4 Relationships among total B_{12}, extracellular B_{12}, B_{12} uptake and the extracellular B_{12} fraction among B_{12} synthesizers (> 50 pM total B_{12}). (A) Extracellular versus total B_{12}. (B) Extracellular B_{12} versus uptake from an initial concentration of 500 pM. (C) Extracellular B_{12} fraction versus uptake. Points represent individual strains. Error bars for total B_{12}, extracellular B_{12} and extracellular B_{12} fraction show geometric standard deviations, whereas uptake error bars show arithmetic standard deviations, calculated from up to four measurements comprising two biological replicates at two time points. Lines show linear-regression fits on the plotted scales, and shaded regions show 95% confidence intervals. Pearson's r and the P-value for the null hypothesis $r = 0$ are shown at the top of each panel.

bioRxiv preprint doi: https://doi.org/10.64898/2026.09.01.748612; this version posted October 14, 2026. The copyright holder for this preprint (which was not certified by peer review) is the author/funder, who has granted bioRxiv a license to display the preprint in perpetuity. It is made available under a CC-BY 4.0 International license.

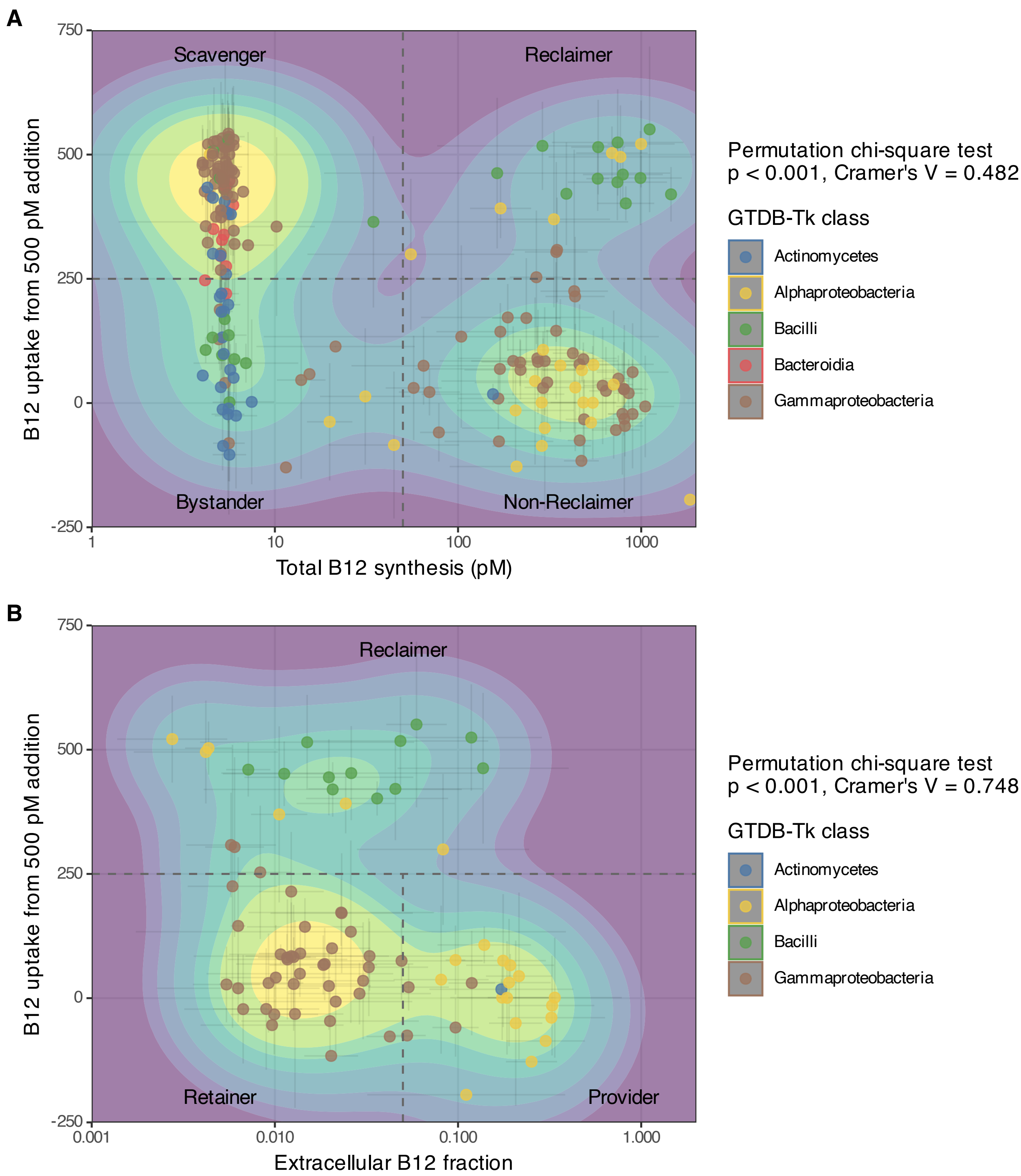

Fig. S5 B_{12} trait space across bacterial taxonomic classes. (A) Total B_{12} versus uptake from an initial concentration of 500 pM for all strains with sufficient measurements. Dashed lines indicate the thresholds used to distinguish bystanders, scavengers, reclaimers, and non-reclaimers: 50 pM total B_{12} and 250 pM uptake. Trait-group membership was associated with GTDB-Tk class (permutation chi-square test with 9,999 permutations: $p < 0.001$, Cramér's $V = 0.482$). (B) Extracellular B_{12} fraction versus uptake among B_{12} synthesizers (> 50 pM total B_{12}). Dashed lines indicate the thresholds used to distinguish retainers, providers, and reclaimers: an extracellular B_{12} fraction of 0.05 and uptake of 250 pM. Trait-group membership was associated with GTDB-Tk class (permutation chi-square test with 9,999 permutations: $p < 0.001$, Cramér's $V = 0.748$). Points represent individual strains and are coloured by GTDB-Tk class; background shading represents normalized two-dimensional point density. Strains without an assigned GTDB-Tk class are shown but were excluded from the contingency-table analyses. Error bars for total B_{12} and extracellular B_{12} fraction show geometric standard deviations, whereas uptake error bars show arithmetic standard deviations, calculated from up to four measurements comprising two biological replicates at two time points.

bioRxiv preprint doi: https://doi.org/10.64898/2026.09.01.748612; this version posted October 14, 2026. The copyright holder for this preprint (which was not certified by peer review) is the author/funder, who has granted bioRxiv a license to display the preprint in perpetuity. It is made available under a CC-BY 4.0 International license.

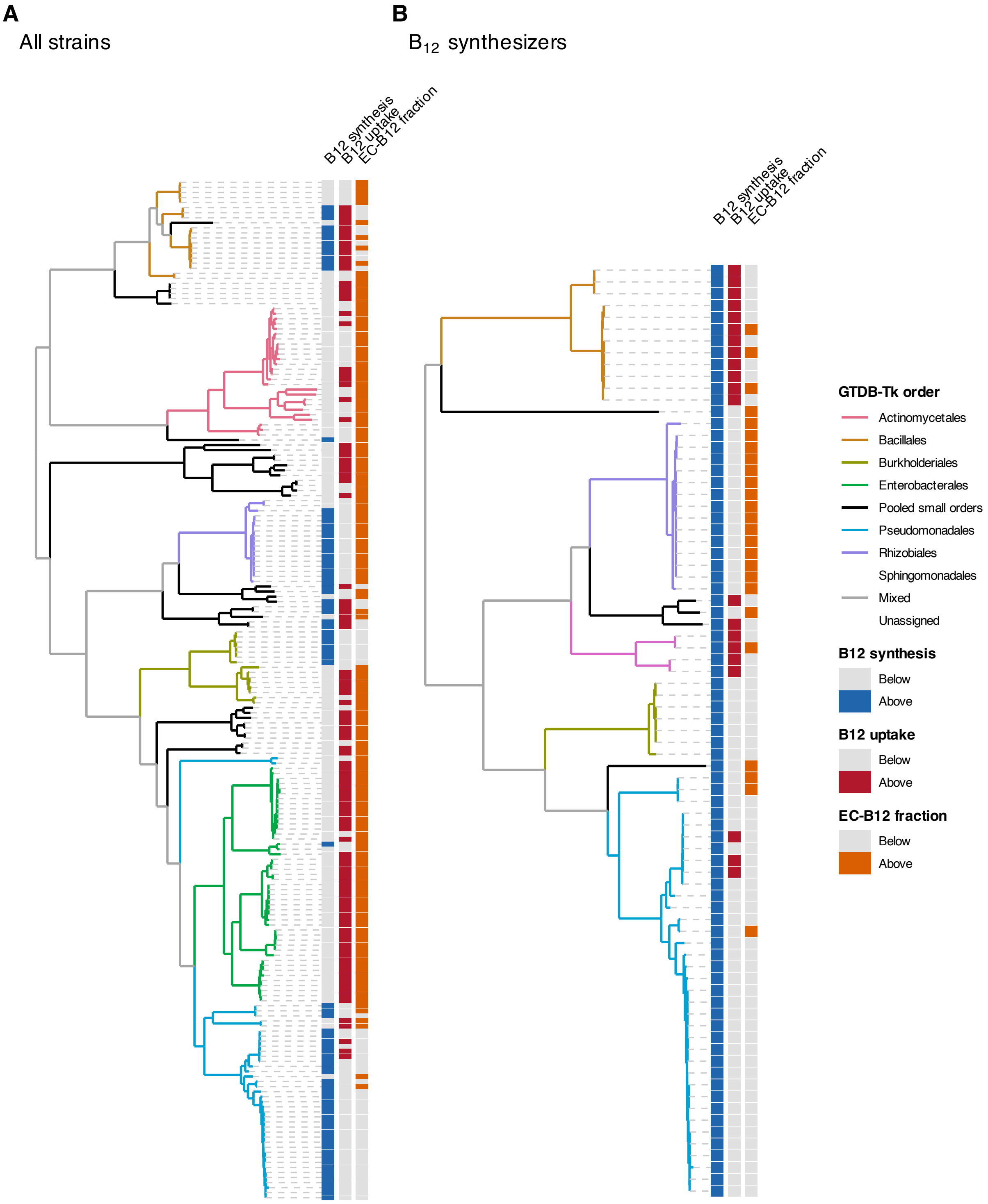

Fig. S6 Phylogenetic distribution of binary B_{12} phenotypes. (A) Binary traits across strains with available phenotype data. (B) The corresponding tree restricted to B_{12}-synthesizing strains with total $B_{12} > 50$ pM and complete uptake and extracellular-fraction measurements. Traits were classified as above the indicated threshold when total $B_{12} \geq 50$ pM, mean B_{12} uptake ≥ 250 pM, or the extracellular B_{12} fraction ≥ 0.05; all lower values were classified as below the threshold. Both trees were generated by pruning the GTDB-Tk phylogeny shown in Figure S2 to retain the relevant strains. Branch colours denote GTDB order. Orders containing at least 5% of strains in the respective analysis set were represented separately, whereas smaller orders were combined into the pooled smaller-orders group.

bioRxiv preprint doi: https://doi.org/10.64898/2026.09.01.748612; this version posted October 14, 2026. The copyright holder for this preprint (which was not certified by peer review) is the author/funder, who has granted bioRxiv a license to display the preprint in perpetuity. It is made available under a CC-BY 4.0 International license.

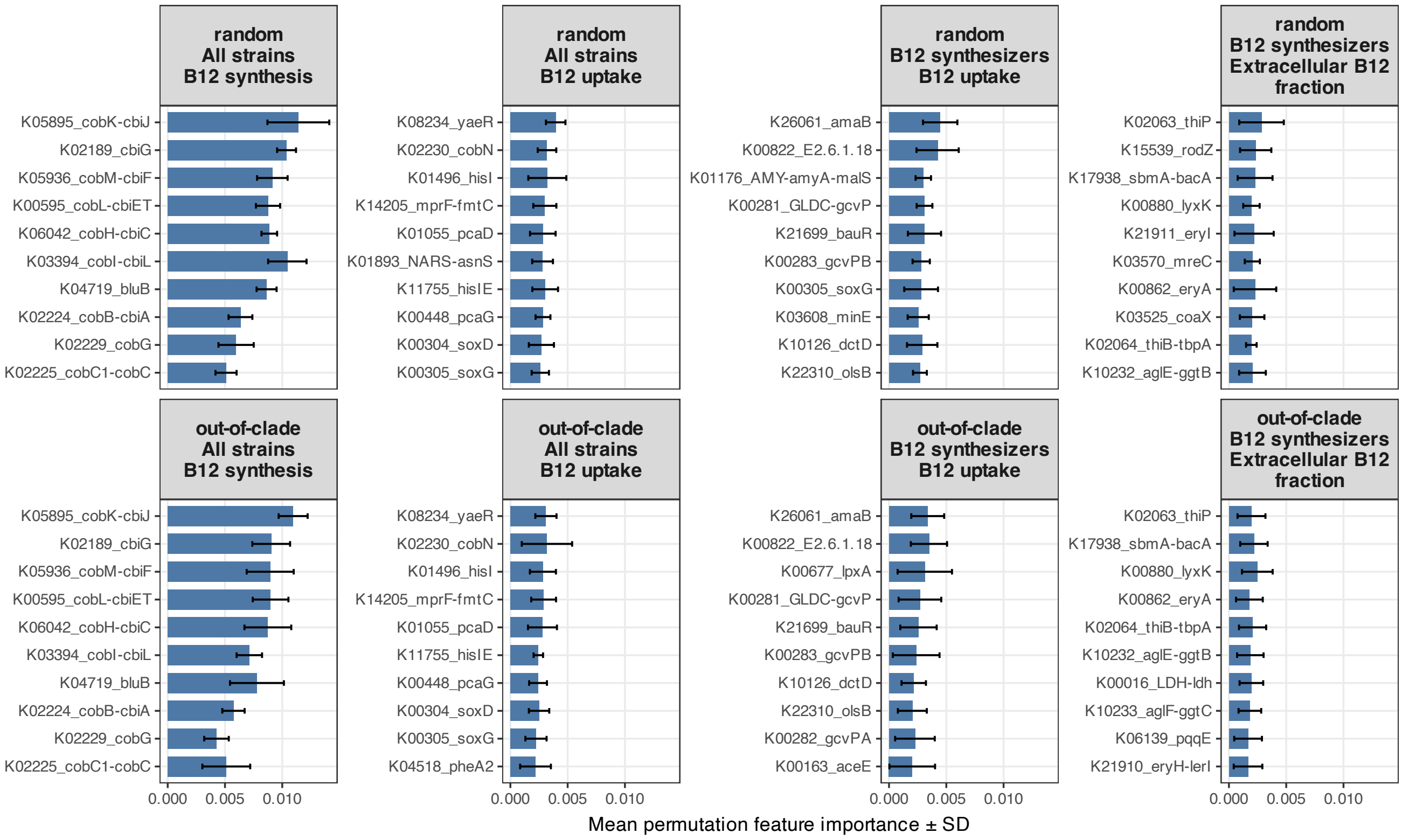

Fig. S7 Most informative genomic features for random-forest classification of B_{12} phenotypes. Mean permutation feature importance is shown for the ten most informative features under random stratified and out-of-clade train–test splits. Models were fitted either to all strains or to B_{12} synthesizers with total $B_{12} > 50$ pM, as indicated. Binary phenotypes were classified as above the threshold when total $B_{12} \geq 50$ pM, mean B_{12} uptake ≥ 250 pM, or the extracellular B_{12} fraction ≥ 0.05; lower values were classified as below the threshold. Error bars represent the standard deviation in permutation importance among model fits.

bioRxiv preprint doi: https://doi.org/10.64898/2026.09.01.748612; this version posted October 14, 2026. The copyright holder for this preprint (which was not certified by peer review) is the author/funder, who has granted bioRxiv a license to display the preprint in perpetuity. It is made available under a CC-BY 4.0 International license.

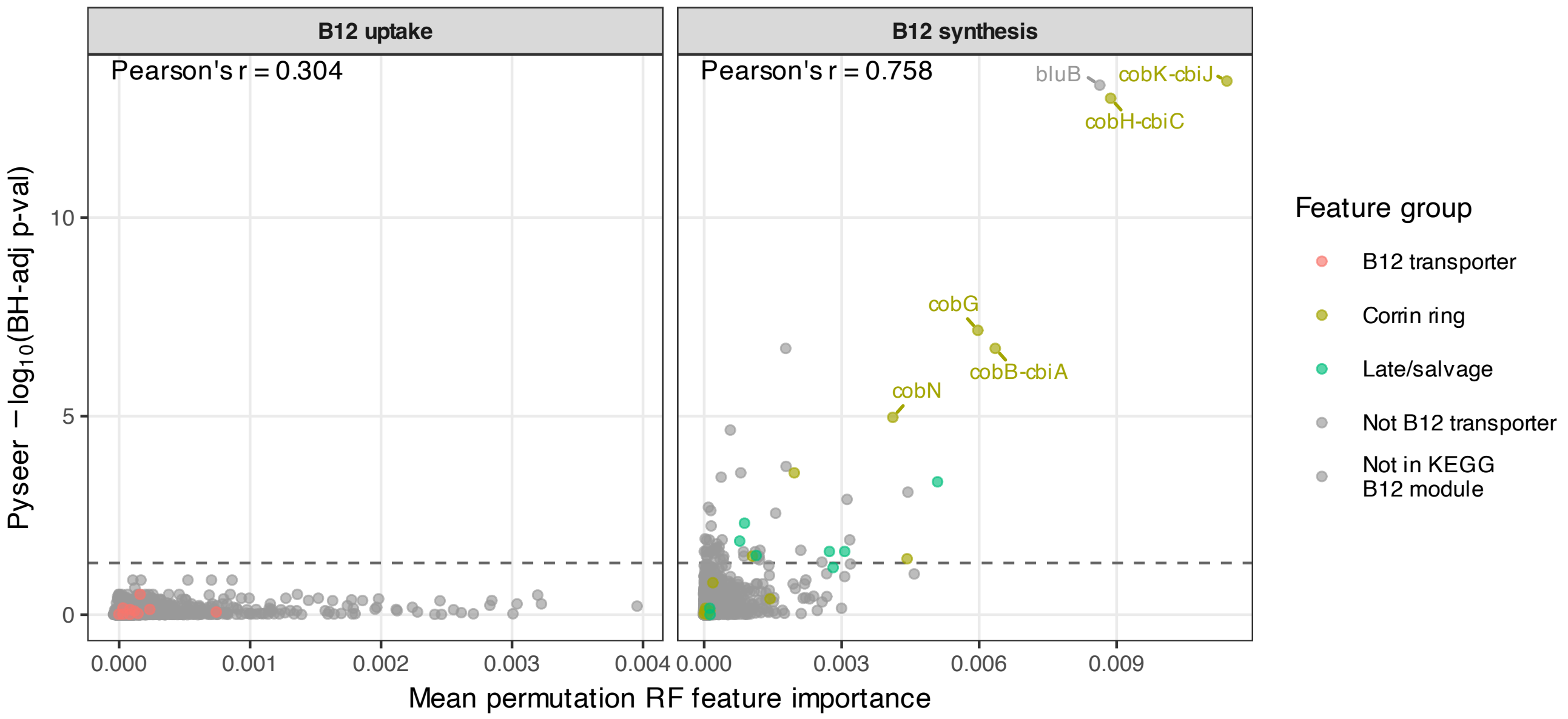

Fig. S8 Comparison of random-forest permutation feature importance with population-structure-adjusted Pyseer association evidence for total B_{12} and uptake phenotypes. Pyseer evidence is shown as $-\log_{10}(P_{\mathrm{BH}})$, where the likelihood-ratio-test P-values were adjusted across all tested KOs for each phenotype using the Benjamini–Hochberg procedure. The 11 candidate B_{12}-transporter KOs were K16092 (*btuB*), K06858 (*btuF*), K25034 (*btuF*), K06073 (*btuC*), K25027 (*btuC*), K06074 (*btuD*), K25028 (*btuD*), K02471 (*bacA/bclA*), K16927 (*cbrT*), K01552 (*ecfA*) and K16785 (*ecfT*). Genes assigned to the corrin-ring category were members of the KEGG anaerobic (M00924) or aerobic (M00925) cobalamin-biosynthesis modules; genes in the late/salvage category were members of the KEGG late cobalamin-completion module (M00122). Although *bluB* (K04719) is not annotated as a member of these KEGG B_{12}-biosynthesis modules, it synthesizes the lower ligand 5,6-dimethylbenzimidazole. Labelled points identify selected annotated genes; grey points denote KOs not assigned to the candidate B_{12}-transporter set or the specified KEGG B_{12} modules. Pearson's r reports the correlation between permutation importance and Pyseer association evidence within each phenotype.

bioRxiv preprint doi: https://doi.org/10.64898/2026.09.01.748612; this version posted October 14, 2026. The copyright holder for this preprint (which was not certified by peer review) is the author/funder, who has granted bioRxiv a license to display the preprint in perpetuity. It is made available under a CC-BY 4.0 International license.

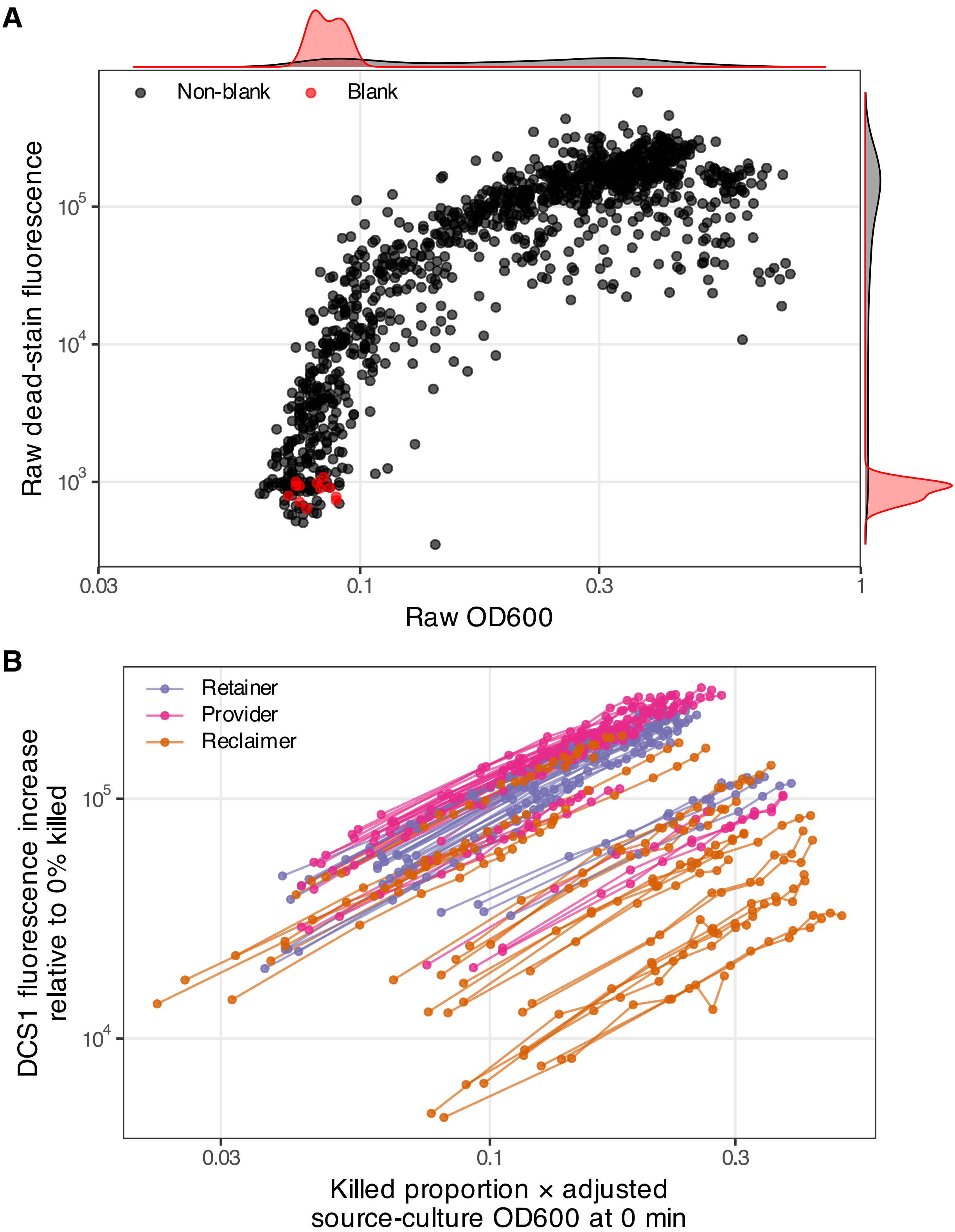

Fig. S9 Validation of dead-cell fluorescence against OD_{600} and controlled live–dead mixtures. Fluorescence was measured using the dead-cell-specific dye Nuclear Green DCS1 (AAT Bioquest; catalogue no. 50-224-9741). (A) Raw OD_{600} before killing versus raw fluorescence after heat killing (95 °C for 30 min) for samples from the bacterial B_{12} trait survey in Fig. 1. Points represent individual culture samples; non-blank samples are black and blanks are red. Marginal densities show the log-transformed OD_{600} and fluorescence distributions. (B) Cultures of 23 selected B_{12}-producing strains were divided into untreated live and heat-killed fractions (95 °C for 30 min), then mixed to produce cultures containing 0, 28.5, 50.5, 66.5, 79, 88, 95 or 100% killed cells. The x-axis shows the experimentally imposed killed-cell fraction multiplied by the blank-adjusted OD of the corresponding source culture at time zero. The y-axis shows the increase in DCS1 fluorescence relative to the matched 0%-killed sample from the same strain and replicate. Lines connect measurements within these matched groups; colours indicate retainer, provider and reclaimer trait groups.

bioRxiv preprint doi: https://doi.org/10.64898/2026.09.01.748612; this version posted October 14, 2026. The copyright holder for this preprint (which was not certified by peer review) is the author/funder, who has granted bioRxiv a license to display the preprint in perpetuity. It is made available under a CC-BY 4.0 International license.

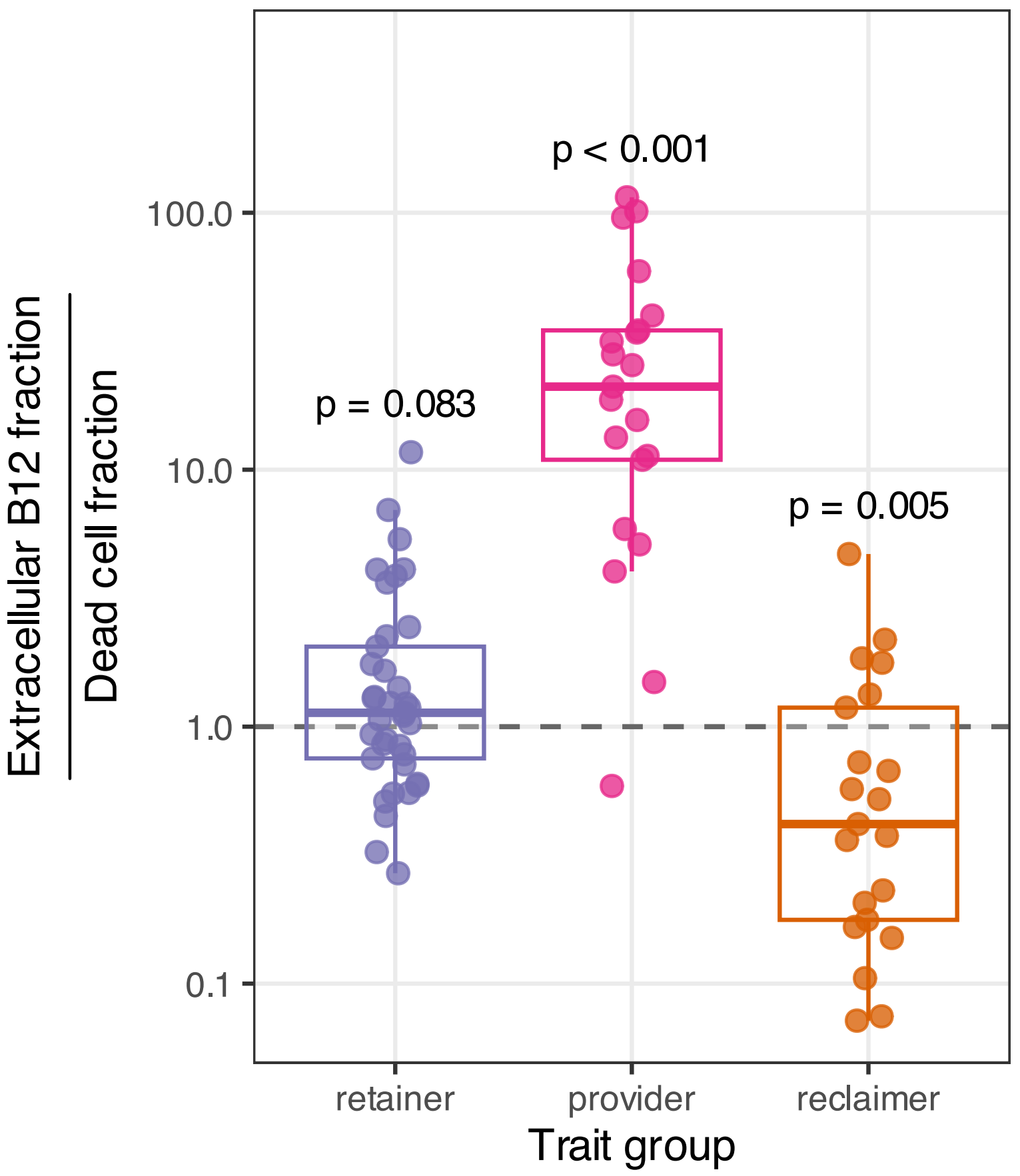

Fig. S10 Extracellular B_{12} fraction normalized by dead-cell fraction across B_{12} trait groups. Points represent strain-level observations, and boxplots summarize the corresponding trait-group distributions. The dashed horizontal line denotes a ratio of one. Within each trait group, the annotated P-value was calculated using a two-sided one-sample Student's t-test of the $\log_{10}$-transformed ratios against zero, corresponding to testing whether the geometric mean untransformed ratio differs from one.

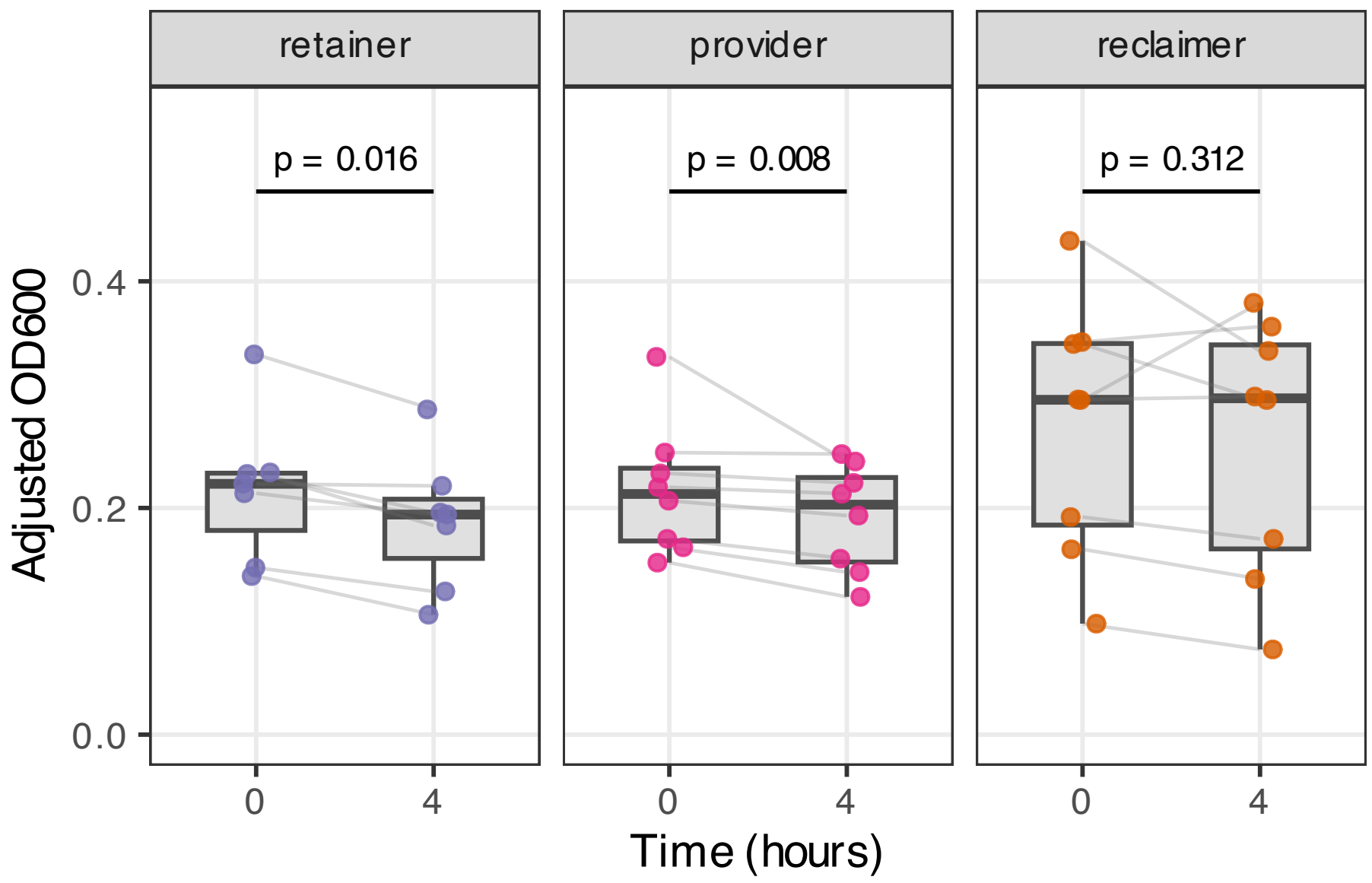

Fig. S11 Initial and final culture density during the B_{12} uptake assay without added glucose. OD_{600} was measured in the source cultures before B_{12} addition and after four hours in the corresponding wells supplied with 500 pM B_{12}. Measurements were blank-subtracted. Points represent strain-level means across four matched cultures, lines connect the same strain, and boxplots summarize the corresponding trait-group distributions. Within each trait group, the annotated P-value was calculated using a two-sided paired Wilcoxon signed-rank test comparing initial and final strain-level OD_{600}.

bioRxiv preprint doi: https://doi.org/10.64898/2026.09.01.748612; this version posted October 14, 2026. The copyright holder for this preprint (which was not certified by peer review) is the author/funder, who has granted bioRxiv a license to display the preprint in perpetuity. It is made available under a CC-BY 4.0 International license.

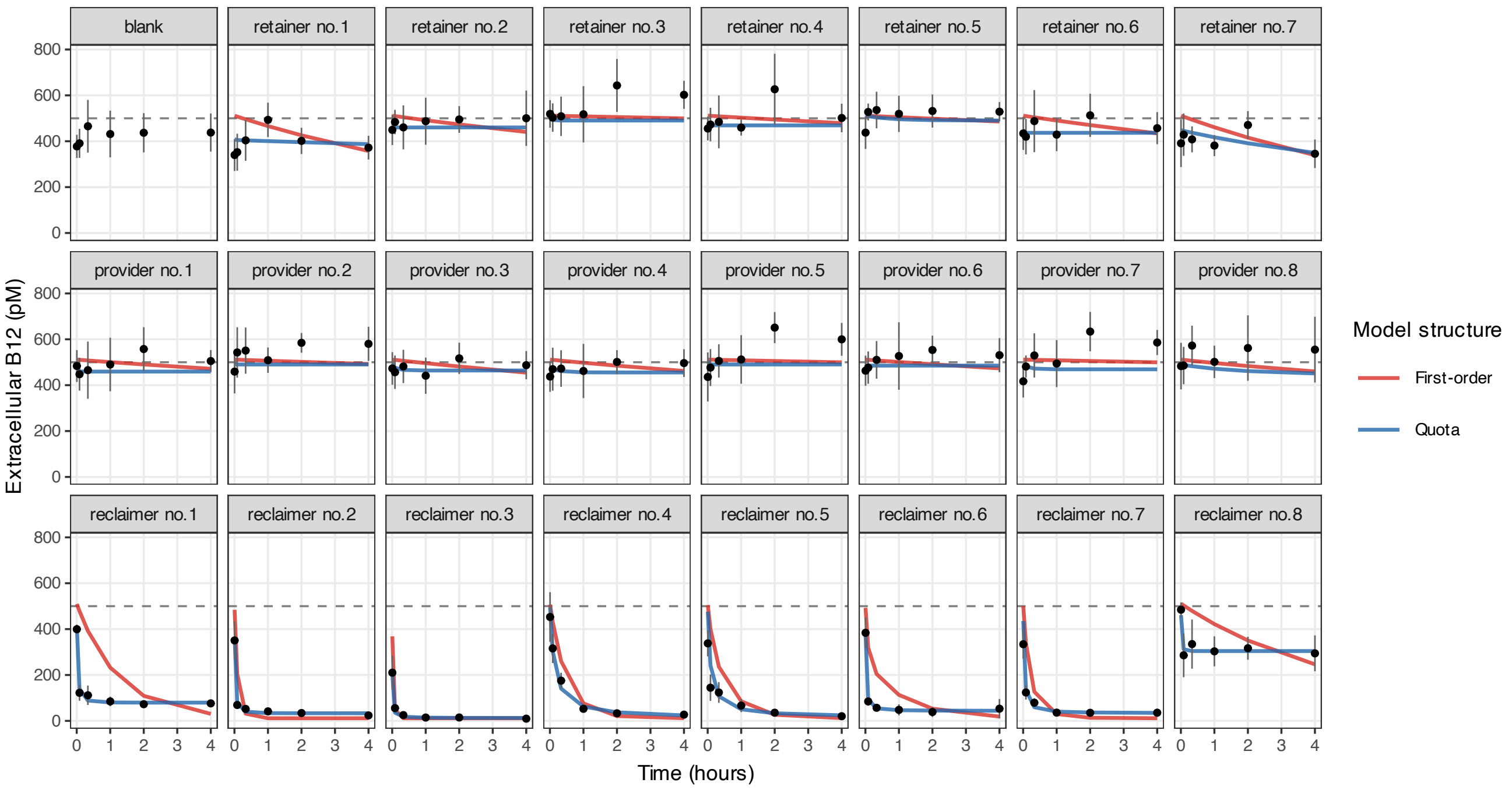

Fig. S12 Empirical extracellular B_{12} measurements and fitted uptake-model predictions in the absence of added glucose. First-order and quota-limited uptake models were fitted to log-transformed extracellular B_{12} concentrations. The concentration at $t = 0$, B_0, was fixed as the sum of the experimentally added B_{12} concentration and the common blank offset, B_{blank}. The OD-specific uptake coefficient k was estimated from the subsequent time course; the quota model additionally fitted an uptake capacity $Q_{\max}$, with uptake slowing as this capacity was filled. Points show empirical measurements; red and blue lines show first-order and quota-model predictions, respectively. Facets show the blank and individual strains ordered within retainer, provider and reclaimer trait groups.

bioRxiv preprint doi: https://doi.org/10.64898/2026.09.01.748612; this version posted October 14, 2026. The copyright holder for this preprint (which was not certified by peer review) is the author/funder, who has granted bioRxiv a license to display the preprint in perpetuity. It is made available under a CC-BY 4.0 International license.

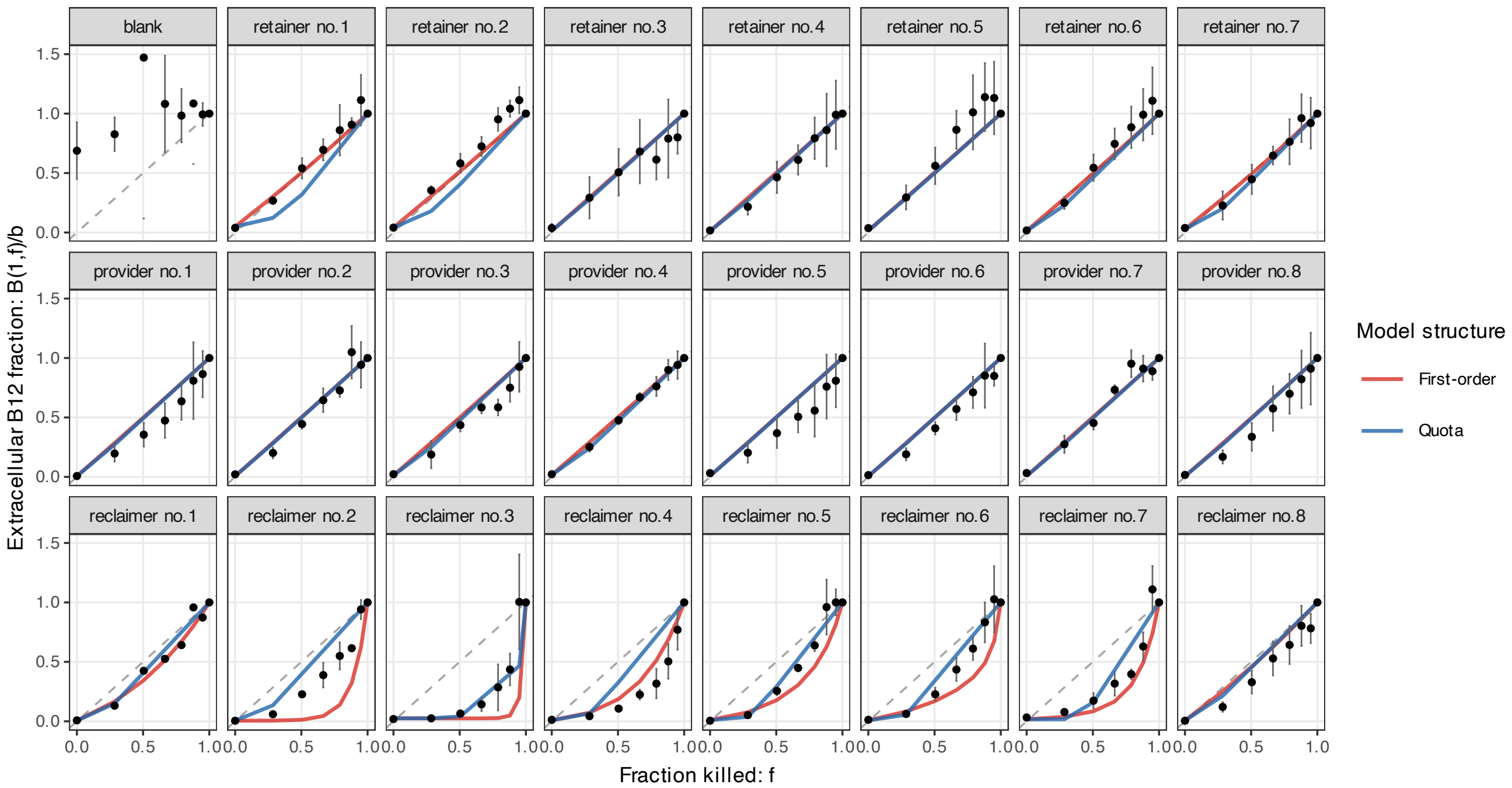

Fig. S13 Prediction of extracellular B_{12} availability in controlled live–dead cell mixtures using independently estimated uptake parameters. For each strain, the first-order and quota-limited models assume that killed cells instantaneously release intracellular B_{12} in proportion to the dead-cell fraction f, while the surviving fraction $1 - f$ recaptures extracellular B_{12} according to the uptake parameters estimated from the corresponding uptake experiment (Fig. S12). Empirical and predicted extracellular B_{12} concentrations after 1 h were normalized to their corresponding empirical and predicted concentrations in the fully heat-killed culture ($f = 1$). Points show normalized empirical measurements; red and blue lines show first-order and quota-model predictions, respectively. The dashed diagonal indicates equality between the extracellular B_{12} fraction and the fraction killed. Facets show the blank and individual strains ordered within retainer, provider and reclaimer trait groups.

bioRxiv preprint doi: https://doi.org/10.64898/2026.09.01.748612; this version posted October 14, 2026. The copyright holder for this preprint (which was not certified by peer review) is the author/funder, who has granted bioRxiv a license to display the preprint in perpetuity. It is made available under a CC-BY 4.0 International license.

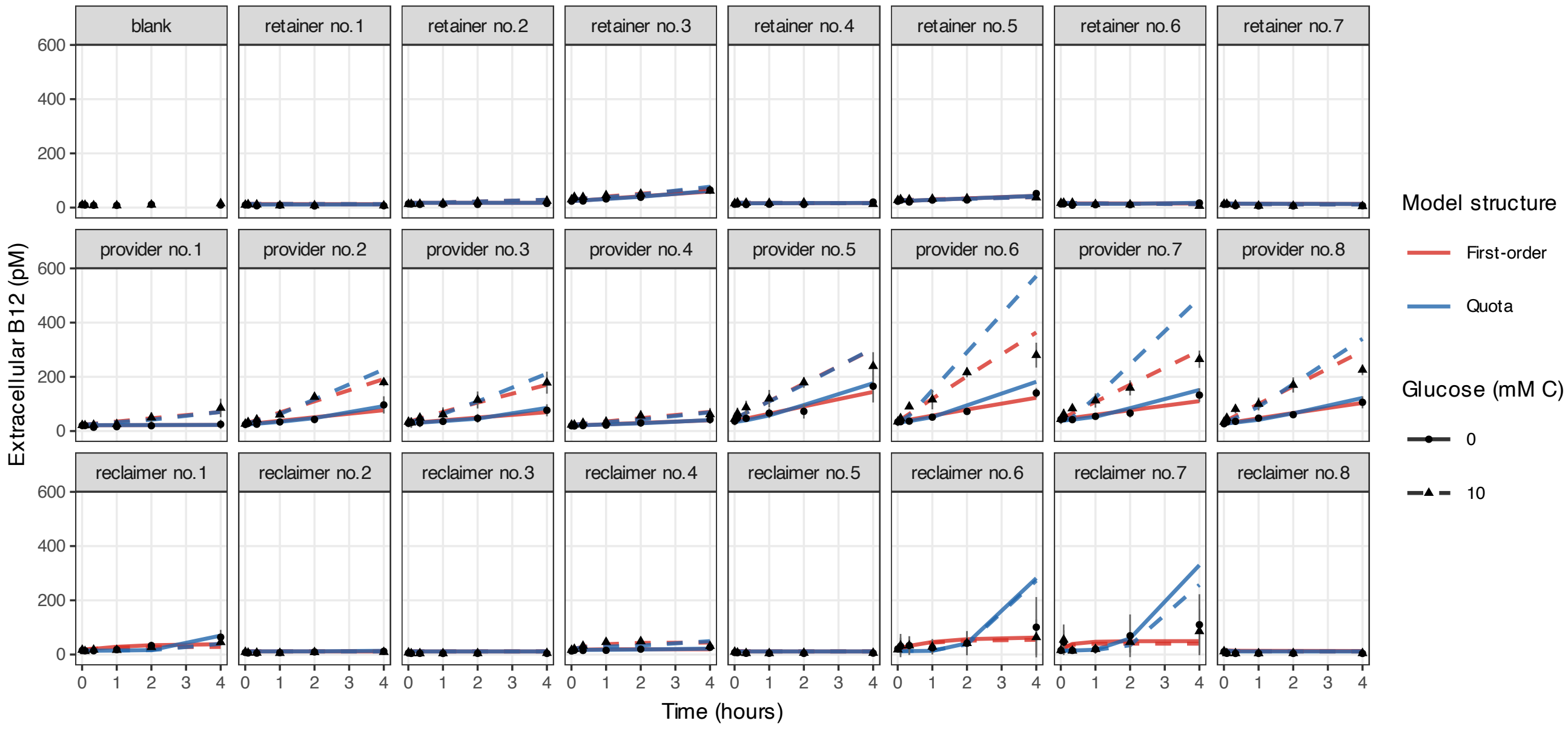

Fig. S14 Empirical extracellular B_{12} measurements and release-model predictions with or without added glucose. Glucose was supplied at 10 mM carbon atoms. Predictions were generated using the first-order and quota-limited models described in Methods. For each model, the corresponding uptake parameters were estimated independently from the uptake experiment and held fixed, while the OD-specific release-rate coefficient r was fitted. The quota model additionally limits cumulative uptake through the fitted capacity $Q_{\max}$. Both models account for culture density using the mean initial and final blank-adjusted OD_{600} and retain the common blank offset. Points show empirical measurements; red and blue lines show first-order and quota-model predictions, respectively. Dashed lines and triangles denote cultures receiving glucose; solid lines and circles denote cultures without added glucose. Facets show the blank and individual strains ordered within retainer, provider and reclaimer trait groups.

bioRxiv preprint doi: https://doi.org/10.64898/2026.09.01.748612; this version posted October 14, 2026. The copyright holder for this preprint (which was not certified by peer review) is the author/funder, who has granted bioRxiv a license to display the preprint in perpetuity. It is made available under a CC-BY 4.0 International license.

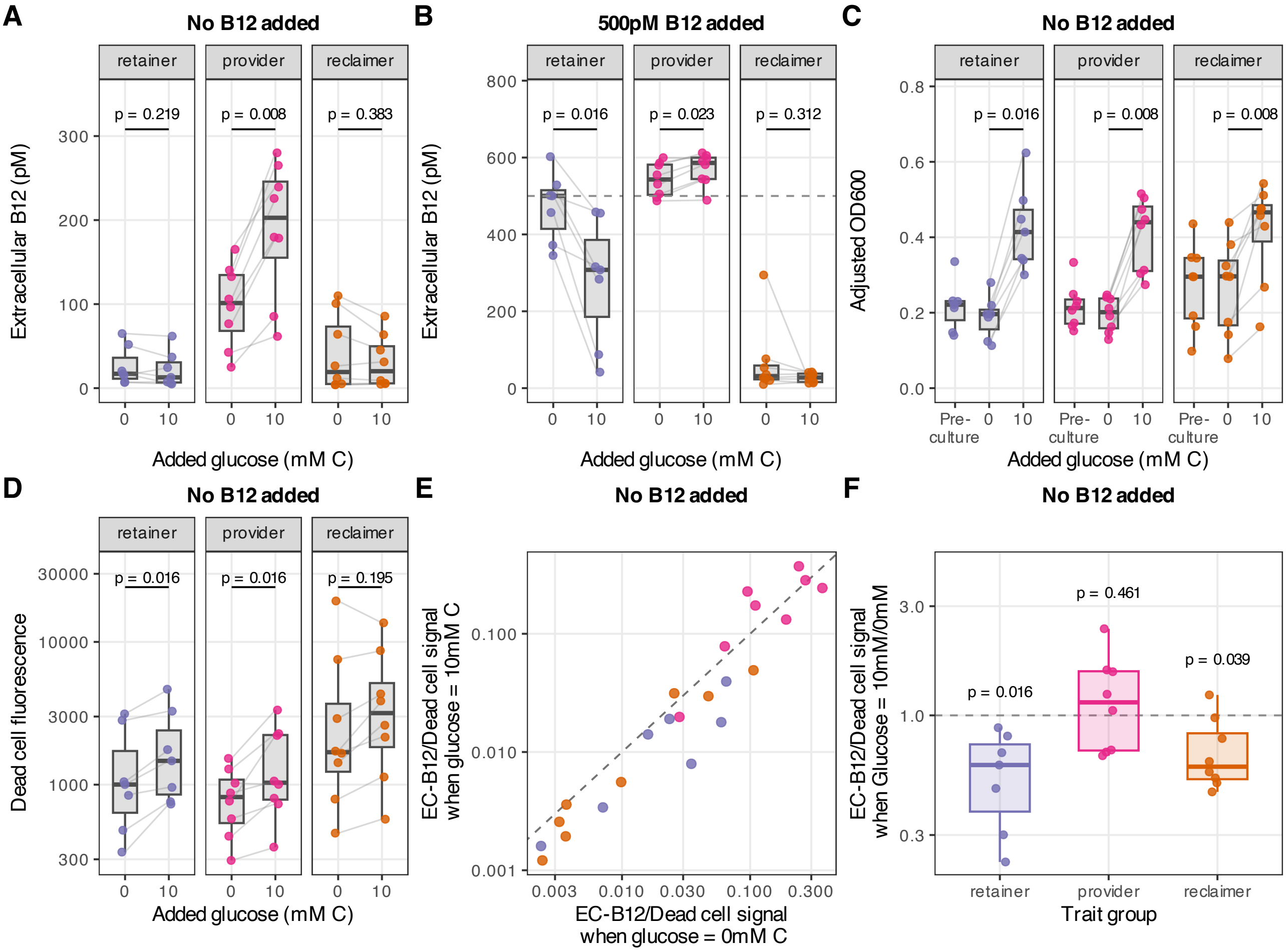

Fig. S15 Effects of added glucose on extracellular B_{12}, culture density and dead-cell signal after 4 h of culturing. Glucose treatments were 0 or 10 mM carbon atoms (1.67 mM glucose). All panels except panel B show cultures without added B_{12}. (A) Extracellular B_{12} without added B_{12}. (B) Extracellular B_{12} following the addition of 500 pM B_{12}; the dashed horizontal line indicates the added concentration. (C) Blank-adjusted OD_{600} before experimental setup (Pre-culture) and after 4 h without added B_{12}, with or without glucose. (D) Dead-cell fluorescence without added B_{12}, shown on a logarithmic scale. In panels A–D, points represent strain-level means, grey lines connect the same strain between glucose treatments and boxplots summarize the distributions. Facets separate retainers, providers and reclaimers. Annotated P-values are from two-sided exact paired Wilcoxon signed-rank tests comparing the two glucose treatments within each trait group. (E) Strain-level extracellular B_{12} (from panel A) normalized by dead-cell signal (from panel D) under 10 versus 0 mM glucose carbon, with both axes shown on logarithmic scales. The dashed diagonal denotes equal normalized extracellular B_{12} under the two treatments. (F) The corresponding within-strain ratio between the 10 and 0 mM glucose-carbon treatments, summarized by trait group; the dashed horizontal line at one denotes no glucose-associated change.

bioRxiv preprint doi: https://doi.org/10.64898/2026.09.01.748612; this version posted October 14, 2026. The copyright holder for this preprint (which was not certified by peer review) is the author/funder, who has granted bioRxiv a license to display the preprint in perpetuity. It is made available under a CC-BY 4.0 International license.

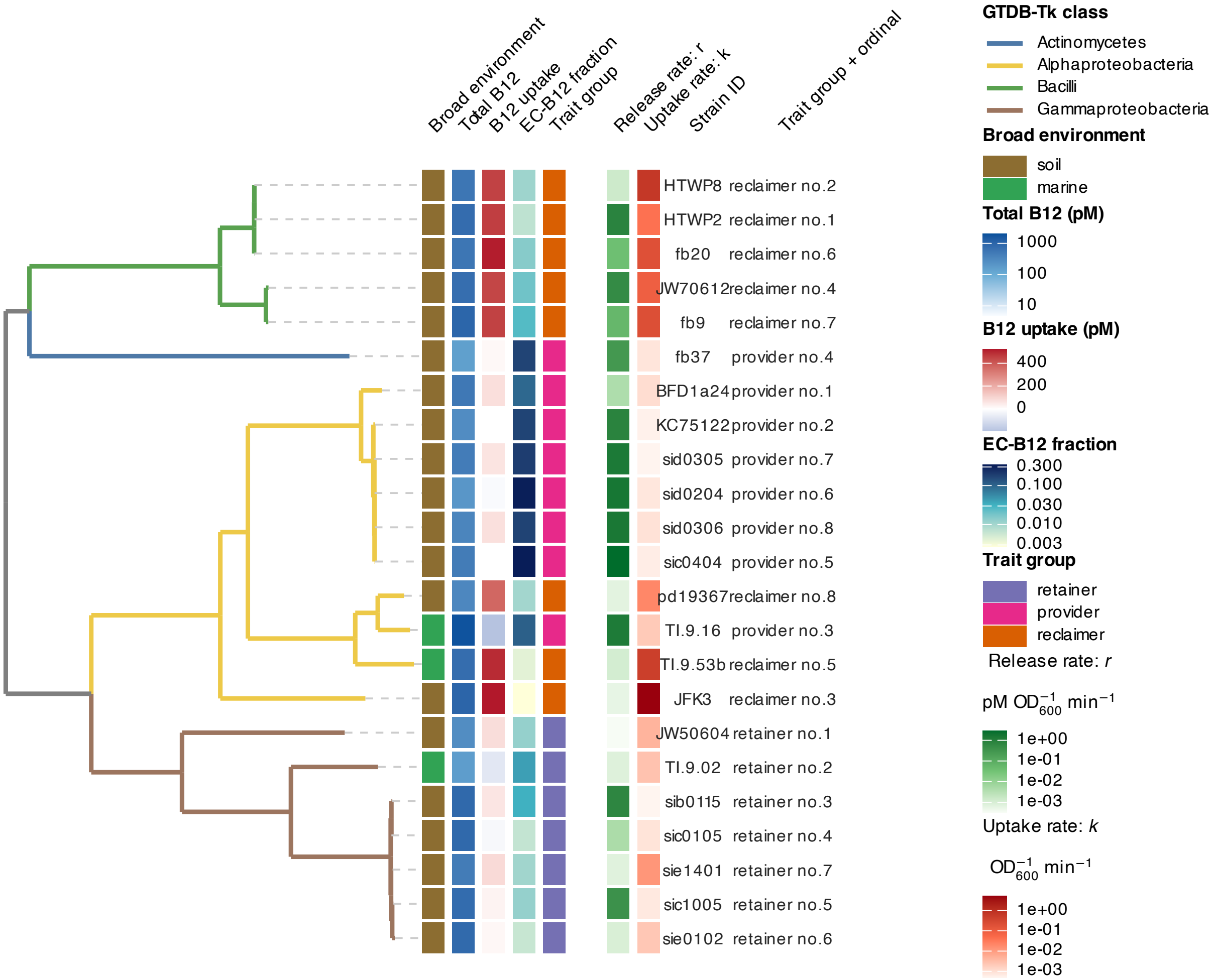

Fig. S16 Phylogenetic distribution of B_{12}-related traits and first-order release and uptake rates among the 23 strains selected for dynamic measurements. The pruned GTDB-Tk phylogeny retains branch lengths, with branch colours indicating taxonomic class and dashed grey lines linking terminal branches to the corresponding data rows. Columns show, from left to right, broad environmental origin; total B_{12}; B_{12} uptake; the extracellular B_{12} fraction (EC-B_{12} fraction); trait group; the OD-specific release-rate coefficient r; the uptake-rate coefficient k; strain identity; and the strain's trait-group ordinal. Total B_{12}, EC-B_{12} fraction, r and k are displayed on logarithmic colour scales. Release and uptake rates are geometric means across biological replicates in the absence of added glucose and were obtained from the first-order models. Trait-group ordinals correspond to those used in Fig. 4, 5 S12, S13, S14.

bioRxiv preprint doi: https://doi.org/10.64898/2026.09.01.748612; this version posted October 14, 2026. The copyright holder for this preprint (which was not certified by peer review) is the author/funder, who has granted bioRxiv a license to display the preprint in perpetuity. It is made available under a CC-BY 4.0 International license.

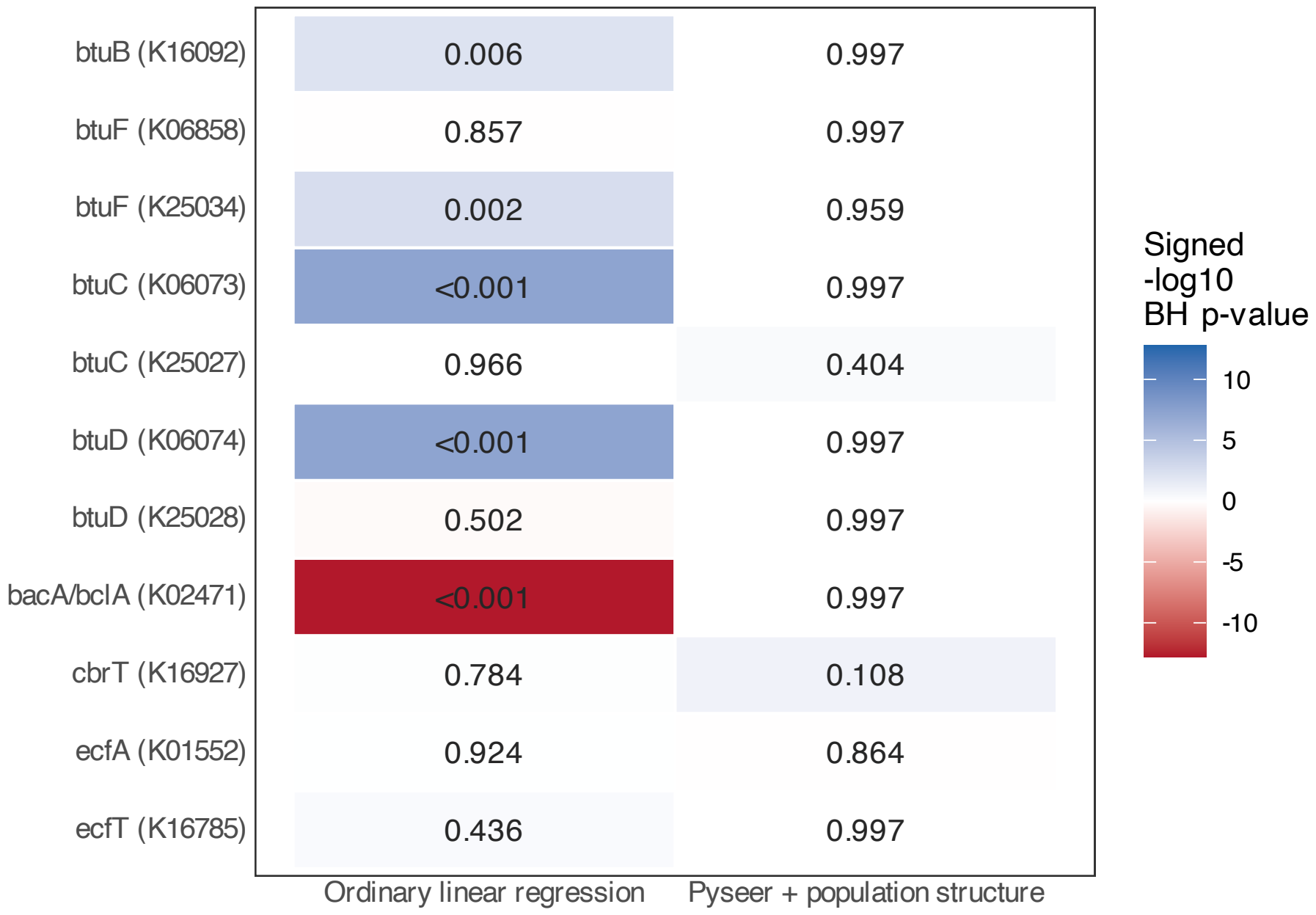

Fig. S17 Associations between candidate B_{12}-transporter KOs and strain-level mean B_{12} uptake. KO copy counts were binarized as absent (count $= 0$) or present (count > 0). For the ordinary linear-regression analysis, mean B_{12} uptake was modelled as a function of this binary indicator; the fitted coefficient therefore represents the difference in mean uptake between strains in which the KO was present and absent. These results were compared with likelihood-ratio tests from Pyseer linear mixed models, which tested the same presence–absence associations while accounting for population structure. Benjamini–Hochberg adjustment was performed across all variable genomic KOs tested by each method, rather than only across the 11 candidate transporters displayed here. Tile colour represents the signed $-\log_{10}$ Benjamini–Hochberg-adjusted P-value, with the sign determined by the fitted association coefficient; values printed within tiles are the adjusted P-values. Some KOs had perfectly correlated presence–absence profiles across strains (btuC-K06073 and btuD-K06074). Pyseer tested one representative from each such cluster, and the representative's coefficient and P-value were assigned to the other cluster members for display; correlated members were not counted as additional independent tests during multiple-testing correction.

bioRxiv preprint doi: https://doi.org/10.64898/2026.09.01.748612; this version posted October 14, 2026. The copyright holder for this preprint (which was not certified by peer review) is the author/funder, who has granted bioRxiv a license to display the preprint in perpetuity. It is made available under a CC-BY 4.0 International license.

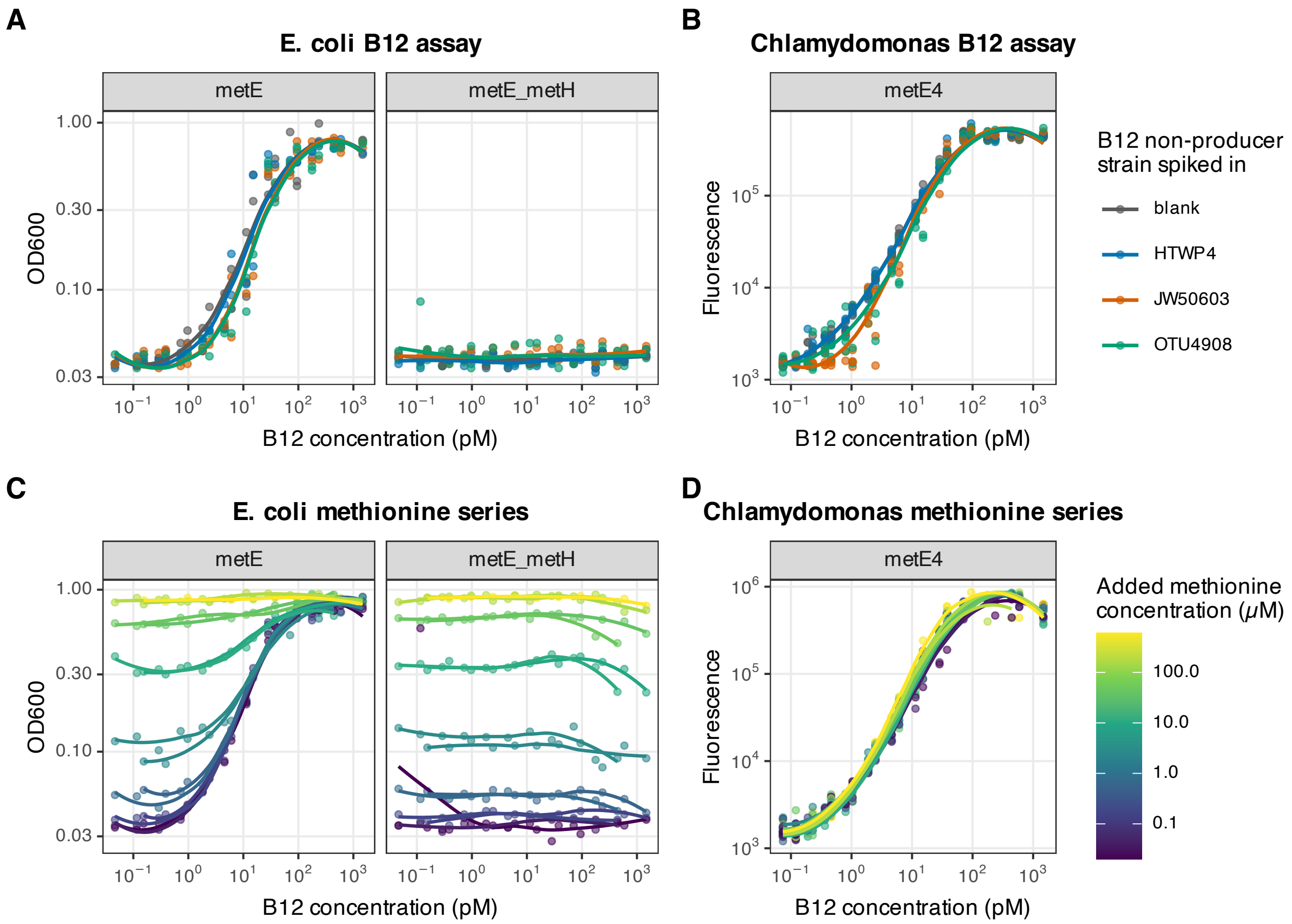

Fig. S18 Comparison of the responses of *Escherichia coli*- and *Chlamydomonas*-based B_{12} bioassays to B_{12}, methionine and bacterial sample matrices. Growth of the *E. coli* assay strains was quantified as optical density at 600 nm, whereas growth of the *Chlamydomonas metE4* assay strain was quantified using chlorophyll-pigment fluorescence, with excitation centred at 400 nm with a 100-nm bandwidth and emission centred at 700 nm with a 100-nm bandwidth. (A) Responses of the *E. coli metE* and *metE metH* strains to B_{12} standards prepared with no added bacteria or with the indicated non-B_{12}-producing bacterial strain. (B) Response of *Chlamydomonas metE4* to the same B_{12} concentration series and bacterial treatments. (C,D) Responses of the *E. coli* and *Chlamydomonas* assay strains, respectively, to combinations of B_{12} and methionine in the absence of added bacteria; colour denotes the added methionine concentration. The *E. coli metE* strain can be supported by either B_{12}, through the remaining B_{12}-dependent methionine synthase MetH, or by exogenous methionine, whereas the *metE metH* double mutant responds only to methionine. In contrast, *Chlamydomonas metE4* responds to B_{12} but not to exogenous methionine. Points show individual measurements and lines show locally fitted response curves.

bioRxiv preprint doi: https://doi.org/10.64898/2026.09.01.748612; this version posted October 14, 2026. The copyright holder for this preprint (which was not certified by peer review) is the author/funder, who has granted bioRxiv a license to display the preprint in perpetuity. It is made available under a CC-BY 4.0 International license.

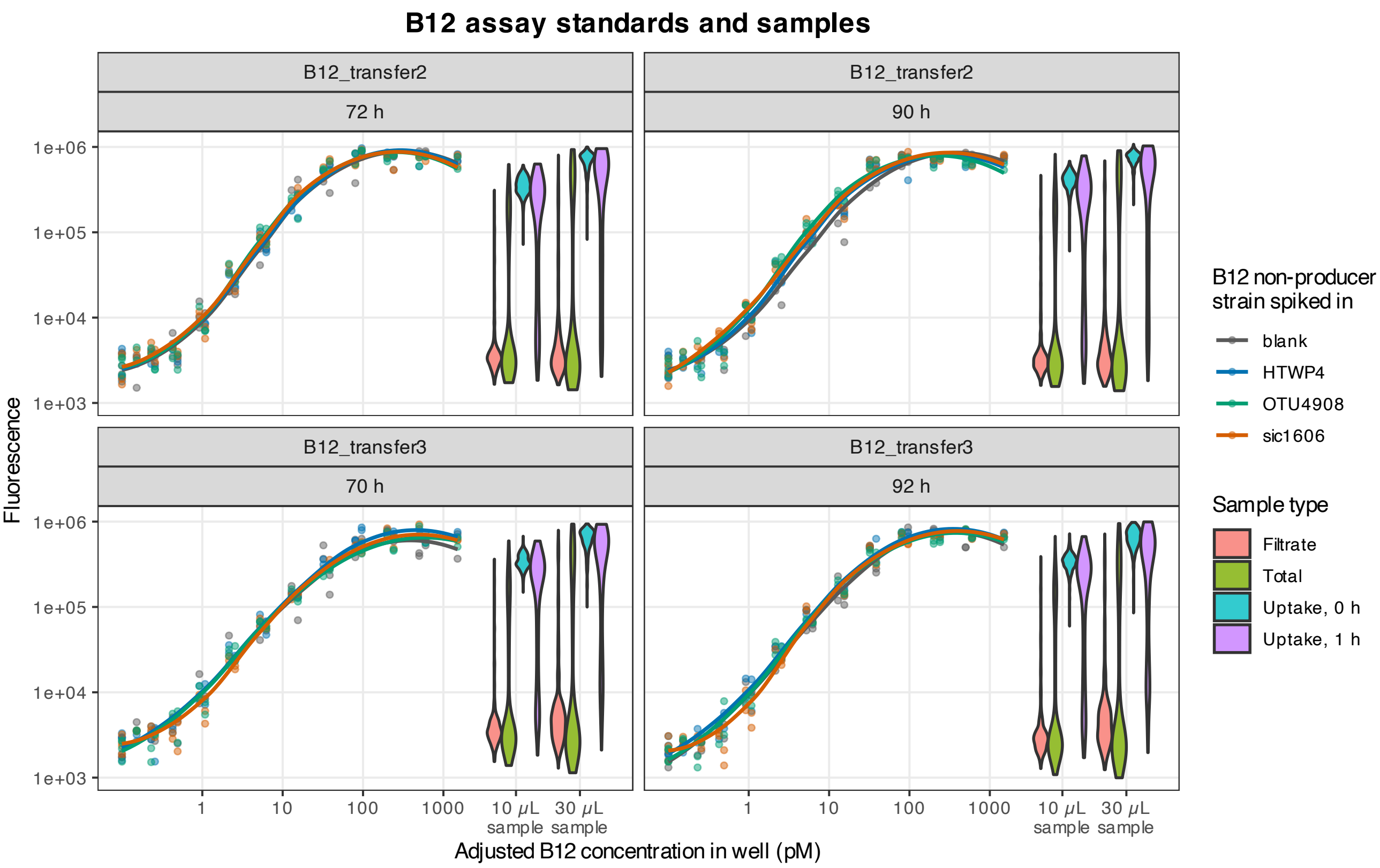

Fig. S19 B_{12}-bioassay standard curves and sample fluorescence distributions for the original B_{12} trait survey of 274 unique bacterial strains, from which the phenotypes analysed in Figures 1–3 were derived. Coloured points and fitted curves show B_{12} standards prepared in blank medium or in culture filtrate from the indicated non-B_{12}-producing bacterial strain, allowing potential sample-matrix effects to be assessed. Violin plots show the fluorescence distributions of filtrate, total-culture, uptake-baseline and uptake-after-1-h samples. Each sample was assayed at two input volumes, 10 and 30 μL, in a final assay volume of 100 μL. The top and bottom facet rows represent two independent experimental replicates. The left and right columns show the two measurement times for each B_{12} assay: 72 and 90 h for the first replicate and 70 and 92 h for the second replicate, respectively. Both axes are shown on logarithmic scales.
